# Supplementary material for: Memory reactivation during sleep does not act holistically on object memory
Source: bioRxiv. 2023 Dec 14:2023.12.14.571683. Preprint. [Version 1] doi: 10.1101/2023.12.14.571683 (PMC10760132; doi:10.1101/2023.12.14.571683)
Supplement: Supplementary file 1 [file NIHPP2023.12.14.571683v1-supplement-1.pdf]

bioRxiv preprint doi: https://doi.org/10.1101/2023.12.14.571683; this version posted December 14, 2023. The copyright holder for this preprint (which was not certified by peer review) is the author/funder, who has granted bioRxiv a license to display the preprint in perpetuity. It is made available under aCC-BY 4.0 International license.

SUPPLEMENT

Table S1:

	Total Sleep	N1	N2	N3	REM
Mean minutes of sleep (SD)	49.26 (18.00)	13.88 (9.73)	21.34 (11.30)	13.06 (14.08)	.99 (3.44)
Mean proportion of sleep (SD)	NA	.31 (.22)	.44 (.19)	.24 (.21)	.02 (.06)
Mean # of cues (SD)	52.82 (28.41)	.12 (.33)	19.83 (21.19)	32.62 (26.89)	0.00 (0.00)
Mean proportion of cues (SD)	NA	.03 (.10)	.45 (.37)	.50 (.39)	.00 (.00)

Note. All information in this table is based on sleep scoring where stages are determined in 30 s epochs. All cues marked in epochs of N1 or wake were followed up and confirmed to occur during N2 or N3 and just prior to a change in stage to N1 or wake. Analyses on effects of sleep stage were performed with these cueing edge cases excluded.
